# Performance-based financing as a health system reform: mapping the key dimensions for monitoring and evaluation

**DOI:** 10.1186/1472-6963-13-367

**Published:** 2013-09-29

**Authors:** Sophie Witter, Jurrien Toonen, Bruno Meessen, Jean Kagubare, György Fritsche, Kelsey Vaughan

**Affiliations:** 1 ReBUILD programme, Queen Margaret University, Edinburgh, Scotland; 2 Royal Tropical Institute, Universal Health Coverage, Amsterdam, Netherlands; 3 Health Financing Unit, Department of Public Health, Institute of Tropical Medicine, Antwerp, Belgium; 4 Health Care Financing, Management Sciences for Health, Cambridge, MA, USA; 5 Africa Region, World Bank, Washington DC, USA; 6 Royal Tropical Institute, Amsterdam, Netherlands

**Keywords:** Performance-based financing, Health systems reforms, Monitoring and evaluation

## Abstract

**Background:**

Performance-based financing is increasingly being applied in a variety of contexts, with the expectation that it can improve the performance of health systems. However, while there is a growing literature on implementation issues and effects on outputs, there has been relatively little focus on interactions between PBF and health systems and how these should be studied. This paper aims to contribute to filling that gap by developing a framework for assessing the interactions between PBF and health systems, focusing on low and middle income countries. In doing so, it elaborates a general framework for monitoring and evaluating health system reforms in general.

**Methods:**

This paper is based on an exploratory literature review and on the work of a group of academics and PBF practitioners. The group developed ideas for the monitoring and evaluation framework through exchange of emails and working documents. Ideas were further refined through discussion at the Health Systems Research symposium in Beijing in October 2012, through comments from members of the online PBF Community of Practice and Beijing participants, and through discussion with PBF experts in Bergen in June 2013.

**Results:**

The paper starts with a discussion of definitions, to clarify the core concept of PBF and how the different terms are used. It then develops a framework for monitoring its interactions with the health system, structured around five domains of context, the development process, design, implementation and effects. Some of the key questions for monitoring and evaluation are highlighted, and a systematic approach to monitoring effects proposed, structured according to the health system pillars, but also according to inputs, processes and outputs.

**Conclusions:**

The paper lays out a broad framework within which indicators can be prioritised for monitoring and evaluation of PBF or other health system reforms. It highlights the dynamic linkages between the domains and the different pillars. All of these are also framed within inter-sectoral and wider societal contexts. It highlights the importance of differentiating short term and long term effects, and also effects (intended and unintended) at different levels of the health system, and for different sectors and areas of the country. Outstanding work will include using and refining the framework and agreeing on the most important hypotheses to test using it, in relation to PBF but also other purchasing and provider payment reforms, as well as appropriate research methods to use for this task.

## Background

Performance-based financing (PBF) is based on a powerful assumption: that individuals and organizations are motivated to perform better by incentives. It is increasingly being applied in a variety of ways and contexts, with varying objectives, and with the overall expectation that it can improve the performance of health systems. However, while there is a growing body of evidence on the design and implementation of PBF^a^[1-3], as well as some evidence of effectiveness from recent impact evaluations [4,5], published evidence on the interactions between PBF and health systems and how these should be monitored and studied remains very limited, particularly in developing countries.

The first Cochrane systematic review of paying providers for performance in low and middle income countries (LMICs) concluded that ‘almost all dimensions of potential impact remain under-studied, including intended and unintended impact on health outcomes, equity, organisational change, user payments and satisfaction, resource use and staff satisfaction’ [6]. This is an important gap in that PBF is increasingly viewed as an intervention which targets systemic change [7]. The review suggested that ‘evaluations should take a broad perspective and consider wider health systems effects, intended or unintended’ [6].

This paper aims to contribute to filling that gap by developing a framework for assessing the interactions between PBF and health systems and for understanding how different ways of designing and implementing PBF ultimately affect the impact of PBF. In doing so, a broader framework which can be applied to monitoring and evaluating health systems reforms is developed. This is intended to complement other ongoing ventures, such as the monitoring and evaluation toolkit which was recently developed to support more rigorous impact evaluations [8]. It fits with a wider call for more ‘systems thinking’ [9], as well as calls for a knowledge exchange on PBF which includes different stakeholder groups [10].

To date most evaluations of PBF have focused on the question of whether it works, which is understood largely in terms of attributing increases in specific service outputs to performance incentives. While this is a valid question that needs further study, it does not do justice to the range of effects (positive and negative) which such a systemic intervention can have, nor does it address the lack of knowledge about how to better design and implement PBF. The focus of this paper is to map out these wider issues, with the aim of encouraging broader empirical work and more rigorous and consistent monitoring and evaluation in the future, on PBF and related reforms. In doing so, it builds on earlier work to evaluate health financing reforms, such as [11].

The paper starts with a discussion of definitions, to clarify the core concept of PBF and how the different related terms are used. Based on an understanding of how PBF mechanisms might in theory strengthen health systems and using the WHO health system building blocks [12], we then adapt a framework for monitoring its interactions with the health system, providing the reader with some key issues to consider when developing their monitoring and evaluation systems. The final section discusses the research agenda which arises from it.

### Background on PBF – definitions and terminology

The plethora of terms commonly used in this field – results-based financing, performance-based incentives, pay for performance, performance-based contracting [13], conditional cash transfers [14], cash on delivery, and others – can cause confusion. At their heart is a resource transfer which is dependent on some form of performance criteria being met. However, that is a very open definition which could be taken to cover all payments systems.

Building on Musgrove’s work [15], we take *results-based financing, performance-based incentives* and *pay for performance*[16] to be over-arching labels, covering all forms of supply-side and demand-side conditional financing. *Conditional cash transfers* are commonly used to denote payments or near-cash transfers such as vouchers to beneficiaries. Some terms are specific to aid, such as *cash on delivery*[17] or *output-based aid*. *Performance-based contracting* is used when contracts are drawn up with non-state actors, such as non-governmental organizations [18].

This paper focuses on one approach within the group, PBF, which is chosen because it is one of the most common approaches being tested in low and middle income countries at present and, secondly, because it involves a more ambitious change to the organization of health systems. The core features of PBF, as currently practiced in reform packages such as those implemented at scale in Central and West Africa, are summarized as follows:

• They take a supply-side approach, meaning performance-based incentives are earned by service providers

• Payments are targeted at individual health facilities and administrations, often with trickle-down to health workers

• There is most often some split of functions between regulation, purchasing, fund-holding, verification and service delivery, although the practices vary by context

• Payments are linked to outputs, modified by quality indicators

PBF can have a number of objectives [6,7,19], including:

• to increase the allocative efficiency of health services (by encouraging the provision of high priority and cost effective services)

• to increase their technical efficiency (by increasing the productivity of existing resources at facility level such as building, equipment and health staff)

• to improve effectiveness of services by greater attention to quality of care

• to improve both coverage rates and equity of outcomes (for example, by encouraging expansion of services to hard-to-reach groups or allowing facilities to reduce fees)

• to increase accountability to stakeholders, as a goal per se but also as an instrument to increase responsiveness of health services and consolidate the collective commitment to finance it.

The core concept is to promote a results-orientation by linking incentives to desired outputs and encouraging entrepreneurial behavior by staff and managers. This is done by establishing a set of more explicit contractual arrangements between different players. Payments do not reflect real service production costs, but aim at investing in front line services and modifying behavior, while leveraging existing resources in the health system.

According to the definition adopted by the African PBF Community of Practice, in August 2010, PBF ‘applies market forces but seeks to correct market failures to attain health gains’*.* Providers are assumed to have, or must be given and supported in using, the autonomy to be able to respond in a creative way to overcome bottlenecks in attaining results at their level. All of these features indicate that PBF is clearly a systems’ change, involving changes in the relationships between actors, structures and processes, as for other provider payment reforms [20]. This means that we need to think systematically about how systems’ arrangements affect the design and practice of PBF, and vice versa.

## Methods

This paper is based on an exploratory literature review focused on monitoring PBF in relation to health systems in LMICs and on the work of a group of academics and PBF practitioners. The group developed ideas for the monitoring and evaluation framework through exchange of emails, working documents and discussion at the Health Systems Research symposium in Beijing in October 2012, where a workshop was held on this topic. It was further refined through circulating a draft for comments to members of the online PBF Community of Practice, which has more than a thousand members (in July 2013), mixing implementers (the largest group), Ministry of Health staff, researchers and international institutional members. 62% of its members are based in low and middle income countries.^b^ A draft was also shared with those who had attended the workshop in Beijing and left their contacts (25 people). Comments from participants were received and incorporated in January 2013. The framework was further presented to a meeting of a group of 35 PBF experts and researchers for comment in Bergen in June 2013.^c^

The literature review built on recent reviews which had been undertaken and on the knowledge of the working group members. The focus was on mapping existing frameworks and evidence for health systems effects of PBF, and what drives them. Few publications on this specific topic were found, though there is an increasing wider literature on PBF. In general, this field of literature is growing but remains largely internal. As noted in a recent review, ‘Of the 100 or so documents reviewed for this paper, only a few were peer-reviewed articles. Many are descriptive briefs or reports by project funders and implementers’ [21]. The framework was therefore developed inductively by the author group, based on their collective experience and a fruitful dialogue between those with experience of designing and operating PBF reform packages with those who have studied them.

## Results

### Adapting a health systems-oriented monitoring and evaluation framework and applying it to PBF

It follows from our definition of PBF that it should, if implemented fully, involve a change in institutional roles and enforcement mechanisms, as well as strengthening of management functions (such as result-based planning and performance management). The interaction of changed incentives, sanctions, institutional changes and supporting mechanisms with existing intrinsic and extrinsic motivation of providers will determine any behavioral changes, which in turn will affect organizational and health systems performance [22].

This starts to provide some of the key nodes which should be reflected in health systems monitoring and evaluation. These should include, at a minimum:

• Initial endowments (roles of actors, their access to information, degree of autonomy, areas of negotiation and uncertainty, rules and norms, access to resources, power relationships, accountability etc.)

• Initial performance of actors at different levels

• Changes to both the initial endowments and the initial performance and factors driving that - again

• What the initial theory of change is, in relation to PBF, and how that is modified as implementation proceeds

Assessments of these should be based on health systems data but also incorporate the views of different stakeholder groups. The theory of change is commonly elaborated during the programme development process, articulating how the intervention is expected to act on key agents and institutions and through what mechanisms and interactions it is expected to generate positive change.

**Figure 1:**
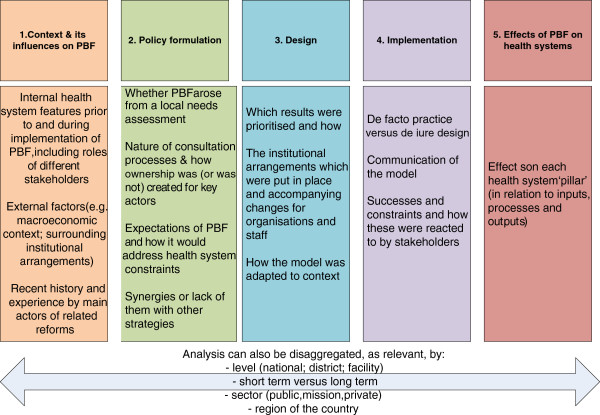
The five domains for understanding PBF-health systems interactions.

A systems perspective implies a dynamic perspective, focusing not just on one-way effects, but interactions, feedback loops and also changes over time. The purpose is not just to document changes but also understand why they happened (or did not happen) and what were the underlying mechanisms and contextual factors of significance. Recognising that health systems are complex adaptive systems is especially relevant for PBF, as for other health system reforms [23]. We argue that this requires an understanding and documentation of five domains (Figure 1), and the continuous interactions between them over time, which will feed back into the underlying theory of change for PBF (what it is aiming to change, and how that is expected to occur).

#### Context

It is clear that the starting conditions in the health system and more broadly will be crucial to whether and how PBF is implemented, and that PBF in turn may have an important influence on those conditions. These context factors operate at organizational, local, national and international levels, and may be enabling or disabling, in relation to PBF (or a combination of both). This section focuses on the influence of context on the operation of PBF reform packages, while in domain five we consider the influence of PBF on the health system.

At the operational level, context includes all elements not under the control of the organizational level which is under scrutiny. Other prior funding streams and access to resources will certainly influence PBF results, such as available input-based funding for recurrent costs or medical supplies like drugs, as well as the volume and uses of user fees. Often these different resource components are reformed at the same time as the introduction of PBF. Other factors which will influence responses to PBF include organizational and management culture amongst providers, and the degree of financial autonomy which they enjoy.

Wider ‘systems software’ [24] – ideas and interests, relationships and power, values and norms – will also affect how PBF is embedded and operated, and its down-stream effects. A strong and stable framework for governance of the health sector may, as in Rwanda, provide the conditions for effective management of a PBF reform package and hold providers to account. Equally, they may make it difficult to develop the necessary split of functions to avoid conflicts of interest in accountability, as interest groups are already entrenched. The converse may be true in fragile states [25-27]. Where facilities are already accustomed to a degree of autonomous financial management, such as under the Bamako Initiative, it may be easier to introduce PBF.

In the wider health sector, it could be argued that there are a number of technical factors which will enhance the chances of PBF succeeding, such as the existence of management competences, having the right size and right skills-mix of human resources for health, the availability of medicines/equipment and infrastructure etc. However, some of the PBF success stories come from countries where a number of conditions were not right, such as Burundi [25,26]. It may be that these worse-off conditions make the creation of new financing and governance structures more urgent, less resisted, and more influential.

The context of health worker pay will also be crucial to the operation of PBF [27] – starting pay levels and conditions, rules governing outside work, sources of external pay, staff management and accountability systems, all of these will directly influence the effects of PBF on staff motivation and behavior.

In terms of the influence of the wider context, PBF is challenged by features such as restrictive public finance rules (e.g. health facilities not being permitted to have their own bank accounts) and underdeveloped banking systems. Indeed, changing some of these constraints may be an outcome of PBF [7]. The macro-economic context is also important: in a growing economy it will be easier to free funds for new initiatives (or to take over initiatives which started as donor-funded pilots). The international environment affects the macro-economic context as well as the availability and conditionality of aid flows.

Domains 2, 3, and 4 cover the three stages of designing and implementing PBF. This is the complex ‘black box’ of what actually evolves on the ground and why.

#### Policy development process

In addition to contextual features, it is important to understand the process through which PBF was introduced, debated, prioritized, crafted and rolled-out. These will influence perceptions of key actors, implementation, and therefore ultimately effects, short and long-term.

Sometimes PBF is introduced or ‘piloted’ by an external financing agency, because these may perceive PBF as increasing their value for money, or prefer to channel their funding directly to the service delivery level [21]. This may lead to hesitations on the host government side, because of fears that PBF may introduce new donor dependency, especially if governments were insufficiently involved in the development process. Governments may however be more open to receive funding in the initial phases of the PBF reform process to invest in capacity building and in structures to create PBF-readiness.

The political interest in and commitment to PBF, and whether it is seen as consistent with or contrary to national priorities, will be critical, as well as whether it is linked with other strategies, such as health worker retention schemes, decentralization, or different approaches to extending health coverage [28].

Other stakeholders’ (national and local, health system and community) perspectives are also important. What were their expectations of PBF? What were the entry points for the introduction of PBF (such as poor health results, or pressure from donors)? Were they involved in its development and in setting priorities within it? What were their interests and experiences in relation to PBF? Did they experience PBF as indigenous or as an idea imposed from outside?

There are also trade-offs to be made between degrees of centralization of the negotiation process. A national level package and pricing allows for consistent priorities to be set, while in a more diverse setting, with greater local capacity, there may be advantages to allowing local level negotiation, to increase ownership of the process.

If PBF is seen as potentially overcoming bottlenecks (internal and external factors that impede performance) in the health system, it will be important to understand what these bottlenecks are (or how they are perceived) prior to the introduction of PBF and how PBF was conceptualized to address these supply- or demand-side problems. This conceptualization is necessary in order to know if and how PBF’s impact on them can be assessed. For each system bottleneck that stakeholders intend to study, it is necessary to identify and describe any other reforms intended to address the same problem. How does PBF reinforce or diverge from other health policies and plans targeting the same problem? [29]? How do they interact? What is the context of related reform experiences in the area?

PBF can be expected to work best when there is underused capacity in the health system, such that limited additional resources can leverage a large increase in outputs. Where this is not the case – where it motivates more activity but the system is already under strain – then the effects on services may be less positive. It is therefore important during the design process to have baseline measures of service production capacity and an understanding of the factors which underlie it.

#### Design features

Domain 3 seeks to understand how different ways of designing PBF ultimately affect its systems effects. There are two main lines of research inquiry regarding PBF design. Firstly, the question of whether the design features and structure work as intended. Do the indicators, targets, and rewards motivate providers to behave as intended? Are the observed changes in provider behavior due to the reward or to the other organizational and operational changes that accompany PBF? The second area of research is looking at how specific PBF features or structures may cause an effect beyond the immediate, intended results.

A key element in the design is defining the priority results to be strengthened through PBF (the size, composition and focus of the service package). Clearly, it is important to understand which services are rewarded, whether they are defined as absolute outputs or as rates (though these are seen as harder to measure) [30], whether they are quantitative only or include quality indicators, and whether the quality indicators (whether relating to processes or outputs) are stand-alone or used to weight quantity indicators. Learning here needs to focus on observing whether one way of structuring indicators and targets works better than the alternatives in motivating the desired behavior in different contexts.

The financial and non-financial incentives attached to the results will also vary, both in their absolute amounts and their potential importance to the recipient organizations, depending on other sources of available financing, on the availability of other inputs, on the cost of doing business and relative pay of health workers. There is no consensus on the proportion of health worker (or facility) pay which should, in theory, be derived from PBF payments in order to motivate better performance. The ‘living wage’ argument would suggest that a higher proportion is justified in contexts where basic pay is low, as is the case in many low income countries. However, there is a contrary argument that small amounts can be motivating when absolute earnings are low. In one study, positive effects were documented with an increase in doctors’ revenue of only 5% [31]. However, in most studies to date in low and middle income countries, the impact on health worker revenues (not to mention behavioural outcomes) has not been documented [21]. This is an area requiring further theoretical and empirical research. The first line question here is whether the amount and payment mode is motivating the recipients to behave as intended.

It is important to understand to what extent the health system context issues, identified in Domain 1, were taken into account in the design of the PBF reform package and its institutional arrangements, including for verification and enforcement. Were PBF arrangements adapted to that context, or were they introduced as a completely new type of health system organization? For example: did PBF use the existing M&E system or was a new data collection system set-up specifically for PBF [29]? Was PBF embedded in the existing governance structure, or was a new PBF structure set-up? How did it affect or build on community voice mechanisms [32]? The first line question here is: is the chosen arrangement working? The broader question is: how does the design of the PBF reform package affect the operation of the wider health system and the responses of key actors? These questions will be linked to the PBF introduction and development process.

In relation to geographic inequity, remote or poorer areas are at a disadvantage to reach targets and receive PBF rewards.^d^ Therefore, some PBF reform packages are designed to have easier targets or adjusted payments that favour less privileged areas (a similar approach to needs-based resource allocation formulae such as that used in the UK). For example, DRC pays 15% higher capitation payments for more remote provinces. Burundi pays 40% higher [25,28,33]. The monitoring and evaluation question here is whether these features do in practice improve geographic equity.

If PBF was set-up as a contracting approach, which usually is the case, it may be evaluated to what extent this influenced later outcomes [34]. If PBF is designed to increase autonomy for providers, transparency of results, and accountability, these are important health system effects to monitor over time.

The distribution of some or all of the PBF resources to staff is another key design feature. It is assumed that PBF affects health systems mainly through changing the attitude and the behavior of staff, but there is still little published research on how that works [21], though some interesting case studies are starting to emerge [35]. The type of incentive (financial or non-financial), amounts, the system for allocating them (and whether this is perceived as fair or not), the other changes to performance management or working conditions which accompany the PBF reforms, all of these – in addition to starting conditions and processes of consultation – will determine how PBF affects staff performance. These may be very different for different types of staff, according to their roles and sectors. Reform packages which do not pass on financial incentives to staff may nevertheless in some contexts achieve good results.^e^

#### Implementation

Under implementation, it is important to compare the theory of the intervention, as laid down in the design, with practice [22]. Changes to design are not necessarily problematic, in that they may be necessary iterative changes, made as a policy hits the reality of the health system. However, it is important to document the actual implementation – how stakeholders were informed of the policy, how their capacity was built to take on new roles, whether funds were disbursed as planned and on time, how verification and counter-verification took place, and so on. The down-stream actions are also very important to whether and how the mechanism works – for example, how did facilities use the resources gained from PBF? How did they adapt their operations in order to respond to the changed incentives introduced by PBF? A case study of performance-based contracting in Uganda illustrates the importance of effective implementation, and also of understanding the emergent behaviours and adaptations of key actors which can take place in response to such intervention [36].

### Health system effects

All health system building blocks are likely to be affected by the introduction of PBF, however for simplicity we have focused on service delivery, human resources, governance and health financing in the framework presented in Table 1. The impacts on commodities are built into the service delivery component, while information systems are regarded as part of the governance function. The effects are organized along a simple results-chain logic – moving from inputs to processes for organizing them, then outputs (and back again – there are strong linkages in both directions, as well as between the pillars). The output changes will be expected to contribute to overall health system goals, including improved health outcomes.

**Table 1:** Framework for monitoring and evaluating PBF’s health systems effects

**Result chain**	**Service delivery**	**Human resources**	**Governance**	**Health financing**
Inputs	Development of quality assurance/improvement tools (like treatment protocols, scorecards)	Changes to working conditions for staff and staff remuneration	Development of governance capacity & systems – e.g. separation of functions	Volume of funds, relative to other sources (globally, and at facility level); and their predictability and variability over time
	Changes to the availability of necessary infrastructure, medicines and supplies	Any change to central level HRH policies and allocation	Investments in improving information and M&E systems	Costs of related investments
		Changes to training (e.g. on good prescribing and evidence-based treatment protocols)	Changes to participation of external stakeholders – especially those representing demand-side	Effect on other financing sources, as relevant. Changes to funds reaching front-line providers
Processes	Changes to organization of services -Effects on quality and convenience for users (“acceptability”). Effects on availability of services, including support services, like diagnostics, lab tests	Changes to availability, retention and distribution of staff (of different types). Change to staff motivation, job satisfaction, teamwork and working patterns, and skills sets	Changes in performance management systems at all levels. Changes to accountability, autonomy, organizational culture and contractual obligations of main actors. Development of leadership skills, at different levels	Allocation of funds (across services, facility types and areas) & link to local needs. Changes to transactions costs (including costs of new governance arrangements, monitoring etc.). How funds are used and any knock-on financial effects (e.g. changes to charges for users)
Outputs	Changes to utilization of services (targeted and untargeted). Changes to coverage – absolute and for different socioeconomic groups. Changes to quality of care (cure rates, readmission, detection etc.). Changes to range and type of services (appropriate to local needs or not)	Changes to staff behavior (working hours, absenteeism, dual practice, informal charging etc.). Evidence of changes to responsiveness and quality of care provided by staff	Changes to health data: regularity, reliability, comprehensiveness. Greater (or less) voice for stakeholders, especially patients. Strategic purchasing practiced. Centralisation/decentralization of functions within sector; changed power relationships within system	Changes to technical & allocative efficiency of services. Sustainability of funding mechanisms & their synergies over time. Changes to affordability for users & financial protection – overall and disaggregated
Health system goals	Better health; greater equity in health; financial protection; responsiveness of health system			

Outputs are presented here in a neutral way – as these may be positive as well as negative. Unintended effects, such as perverse effects in service delivery (e.g. changes in services that were not incentivized through PBF), should be anticipated as far as possible and actively monitored. Effects on coverage and affordability should be monitored in absolute terms and for different socioeconomic groups.

Although outputs are presented in separate pillars, it will also be important to look for linkages across different pillars and between levels of the health system, and areas of synergy (or, indeed, tension), as well as positive and negative feedbacks.

## Discussion

We have adapted a framework for monitoring a health system reform, focusing on its application to the introduction of PBF. This provides an interesting case study not only because this topic is very current, with a fast growth in application in a low and middle income countries at present, but also because PBF has potentially widespread effects in relation to resource allocation, purchasing and provider payments, with knock-on implications for all of the health systems blocks. The aim of the framework was to broaden the dialogue around PBF and give a more system-oriented lens for its study. There are however limitations to the approach taken. A recent search found 41 different frameworks for conceptualising health systems [37]. These were grouped into frameworks for understanding health systems, comparing them, informing change, and evaluating it. The authors further grouped the frameworks according to whether they focused on sub-systems, whole systems, or supra-systems (focussing on linkages with other sectors). Within this typology, this paper focuses on the whole system, and in particular on evaluating changes to it. While the wider linkages outside the sector are important, the interactions within the health sector are sufficiently complex that we focus our framework on understanding and monitoring them. Other conceptualisations could have been employed, such as the ‘control knobs’ model [38], which identifies five major “control knobs” of a health system which policymakers can use to achieve health system goals (financing, organization, payment, regulation, and behaviour). PBF interacts strongly with each of these.

Clearly, the choice of indicators to focus on within this framework will be influenced by particular contextual needs - including the motivation for undertaking the monitoring and evaluation, and the design and theory of change of the particular PBF reform package. It is not realistic to be exhaustive. However, it is important to think system-wide and clearly about what might change, and what is a priority to measure (and why).

The majority of PBF evaluations and monitoring systems to date have focused on changes to outputs, in terms of increased delivery of targeted services. However, most other domains in Table 1 have been neglected by researchers. Good practice indicates that important effects (positive or negative) should be identified in advance and then actively monitored. Ex-post observations are less useful than developing a clear theory of change and then using it to evaluate, leading to a later revision and fine-tuning of the theory and implementation.

Although it may seem that some of the elements in the tables are not necessarily connected to PBF – such as training on good clinical practice, for example - other types of inputs do often accompany PBF reform packages, like capacity building through training, mentoring and supervision, and coaching and technical assistance on measurement, use of improved management tools and how to increase results. These are likely to contribute to any results, and so should be viewed as a package. The financial incentives, though central in terms of the definition of PBF, may not be solely or even, in some cases, centrally responsible for such changes as occur. Attribution of effects to any one strand may be complicated, but by clearly documenting the five domains of context, design process, design features, implementation, and health system effects, plausible linkages can be made.^f^

In terms of staff satisfaction, to give one example, it is to be expected that staff are pleased to receive what is, in almost all cases, additional funding, and indeed a number of reports do find this. However, responses are often nuanced, in that the funding comes with increased pressures and studies do not always differentiate responses to levels of pay with responses to the system for allocating it. The pressures which PBF can put on staff may be constructive, or unconstructive (e.g. if targeted results cannot be met because of external constraints). Involvement in setting targets, ability to control the factors which affect those targets, perception that the measurement and reward processes are fair and transparent, and an adequate level of funding of targeted actions are all factors which are likely to improve staff responses [21].

Health systems are not closed, and the health systems framework may prove too narrow to capture all of the effects of PBF. If it affects staff remuneration, it is likely to have impacts beyond the health sector, affecting other public servants’ expectations, for example. Reforms in health may also influence and be influenced by reforms in other sectors [7,39]. However, the health system frame is likely to pick up most and the most significant results, including (especially within the governance aspects) the political economy of reforms – changing influence and benefits to different groups. Political economy analysis can be a useful tool for investigating this.

Its effect over time is another important area for investigation. Rapid improvements were noted during the start up of some PBF reform packages but later payments became integrated with health worker remuneration, which may lead to a leveling off or even a decline in performance over time, if there is no variation in payments [25]. On the other hand, payment per output may avoid the problem of PBF being seen as an ‘acquired right’ [22].

From a funding point of view, sustainability is likely to depend on a combination of government buy-in, coherence with other strategies, good working relations with donors, perceived and documented good results, and continued external support [21]. All on-going PBF packages in LMICs are highly dependent on external support, though in Rwanda and Burundi the government contribution is substantial.

## Conclusions

The paper lays out a broad framework within which indicators can be prioritised for monitoring and evaluation for this and related health system reforms. It encourages policy makers and researchers to go beyond the few targeted indicators of a PBF package or similar reform (e.g. target groups for health insurance) and look at wider and unintended effects and feed-back mechanisms. It highlights the dynamic linkages between context, process of development, design, implementation and effects and between different health system pillars. All of these are framed within inter-sectoral and wider societal contexts too. It highlights the importance of differentiating short term and long term effects, and also effects at different levels of the health system, and for different sectors (public, private, mission etc.) and areas of the country. Unintended effects and also synergies with other strategies are equally important.

Outstanding work, following from this framework, will include the following:

□ Reaching consensus on models and terminology within the field

□ Using and refining the framework for PBF and other health system reforms

□ Discussion of the most important nodes (based on a theory of change) and appropriate indicators and mixed methods research tools to measure them

□ Developing hypotheses that can be tested, on, for example, in what contexts, in response to what types of challenge, with what design, PBF can strengthen the health system (or subcomponents of the health system)

## Endnotes

^a^See the many postings on the RBF website: http://www.rbfhealth.org/rbfhealth/.

^b^See http://www.abdn.ac.uk/femhealth/documents/CoPANEL_Beijing2012.pdf.

^c^See http://e.itg.be/ihp/archives/pbf-workshop-bergen-impressions-observations-junior-researcher/ for a description of this meeting.

^d^A working group on PBF and equity was launched recently - see http://www.healthfinancingafrica.org/3/category/alex%20ergo/1.html.

^e^See, for example, early results from Zimbabwe: http://www.rbfhealth.org/rbfhealth/news/item/657/rural-zimbabwe-no-more-user-fees-women-and-children.

^f^In a linked article, the group plans to explore the question of appropriate research methods to investigate some of these questions.

## Competing interests

The authors declare that they have no competing interests.

## Authors’ contributions

SW led on the drafting and revision of the article, including review of the literature and conceptualisation. JT coordinated the group and contributed to conceptualisation and drafting. BM contributed to drafting and also to coordinating inputs from wider participants and networks. JK and JK contributed to drafting and revisions. KV contributed to the literature review and drafting. All authors read and approved the final manuscript.

## Authors’ information

SW is a health economist who has been working in health financing in low and middle income countries for nearly twenty years. She recently led a systematic review of performance-based funding in low and middle income countries for the Cochrane Collaboration, and has led or contributed to a number of studies of PBF programmes. She is currently working on health system reconstruction post-conflict with the ReBUILD programme.

JT is a medical doctor specializing in community health, and coordinator for Universal Health Coverage, health insurance, and Performance Based Financing at the Royal Tropical Institute (KIT), Amsterdam. He has supported Ghana and Mali in designing and implementing a PBF programme, and coordinated a formative multi-country evaluation of PBF.

BM is an economist; he heads the Health Economics Unit of the Department of Public Health, Institute of Tropical Medicine, Antwerp. His main domain of expertise is health sector reform, health care financing, performance-based financing, and social health protection in low- and middle-income countries. He has contributed to the design and the study of PBF reforms since 2000 and is the lead facilitator of the Performance Based Financing Community of Practice.

GF is a medical doctor with eighteen years’ experience at the operational level in Zambia, Senegal, Afghanistan, Kenya, South Sudan and Rwanda. He has ten years’ experience in designing and implementing Performance Based Financing programmes in Afghanistan, Rwanda, Burundi, Kyrgyzstan and Nigeria. As a Senior Health Specialist for the World Bank in Washington since 2009, he advises governments in appropriate design and implementation issues related to Results-Based Financing programmes.

JK is the Global Technical Lead in the Center for Health Services at Management Sciences for Health (Cambridge, MA). In his over 20 years experience he has managed various health projects and programmes. In Rwanda he served as the Director of the National HIV/AIDS programme and of the Health Planning Department, was Coordinator of the World Bank Health and Population Project, and was involved in the set-up of PBF in Rwanda.

KV is currently a Health Systems Advisor at KIT helping countries work towards universal health coverage with a special emphasis on costing studies. She recently participated in an evaluation of a PBF programme in DRC which focused on the wider effects of PBF.

## Pre-publication history

The pre-publication history for this paper can be accessed here:

http://www.biomedcentral.com/1472-6963/13/367/prepub
